# Supplementary figures and images for: Validating Atlantic salmon (Salmo Salar) scale reading by genetic parent assignment and PIT-tagging
Source: PLoS One. 2025 May 8;20(5):e0316075. doi: 10.1371/journal.pone.0316075 (PMC12061416; doi:10.1371/journal.pone.0316075)

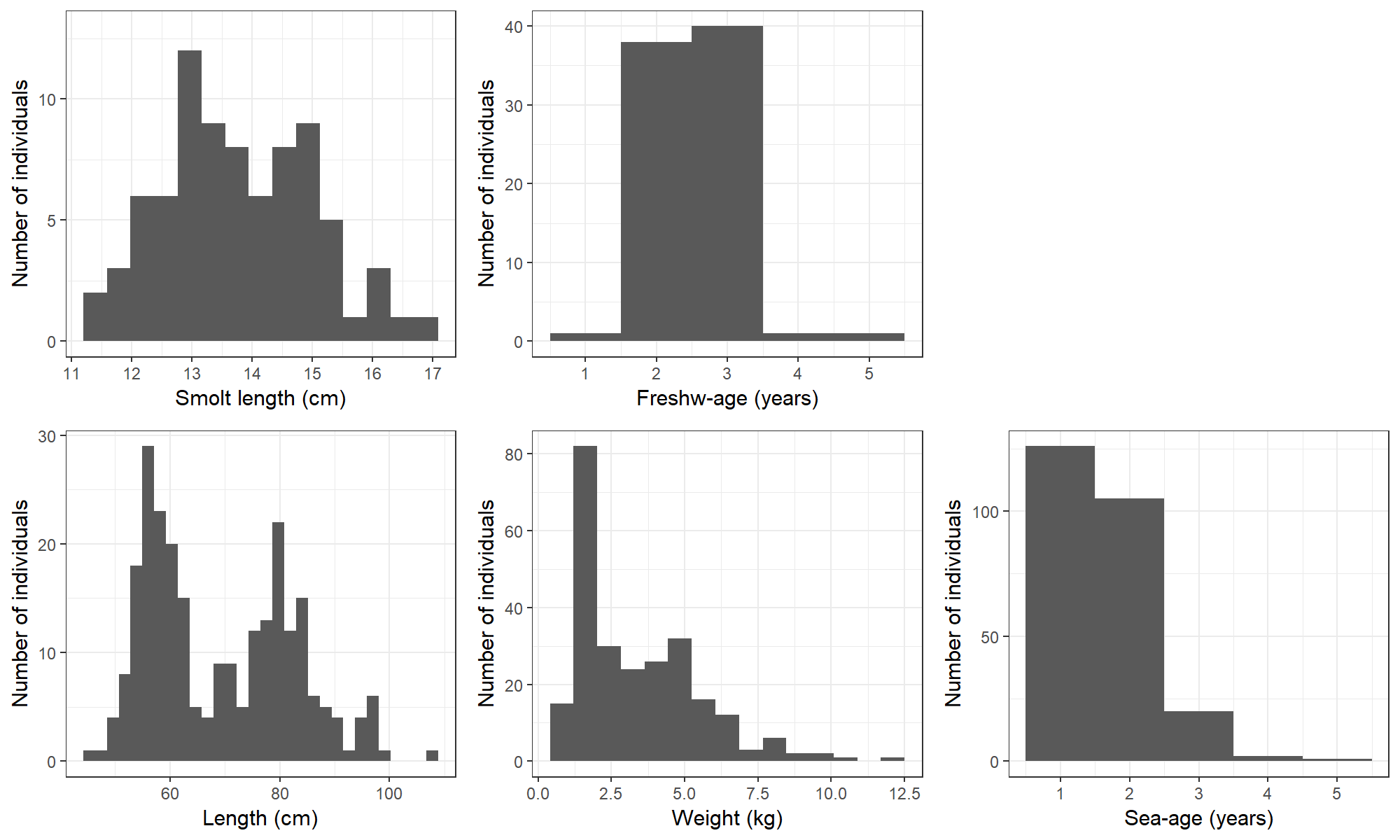

Supplement: S1 Fig — Biological information for the sampled salmon included in this study. A) Measured smolt length, B) True freshwater-age, C) Body-length when returning to the river, D) Body weight when returning to the river, E) True sea-age. (TIFF) [file pone.0316075.s001.tiff]

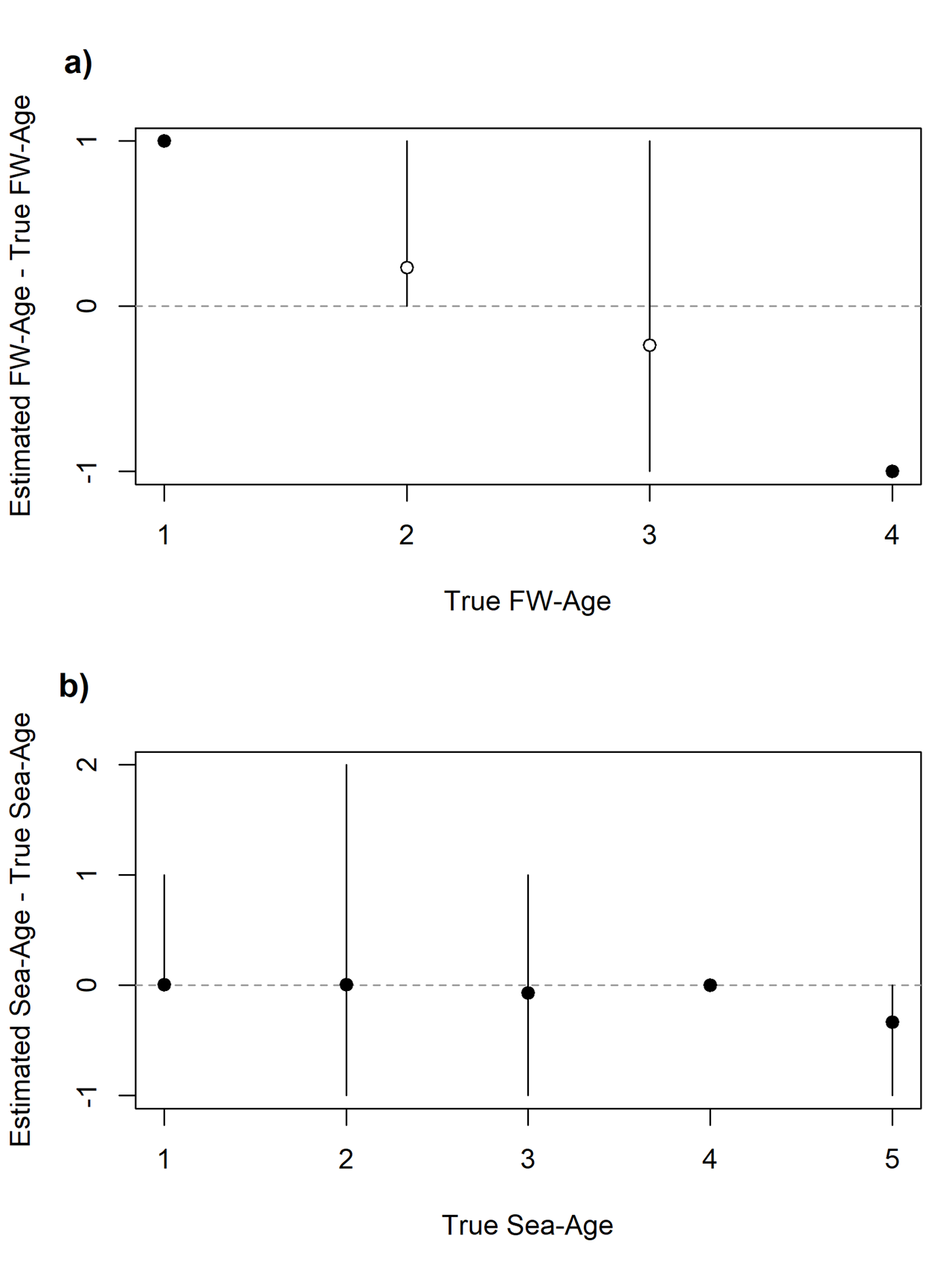

Supplement: S2 Fig — The difference between the true freshwater-age (a) and seawater-age (b) against the corresponding estimates by the scale readers. The circles represent average difference while bars represent the corresponding 95% confidence intervals. (TIF) [file pone.0316075.s004.tif]

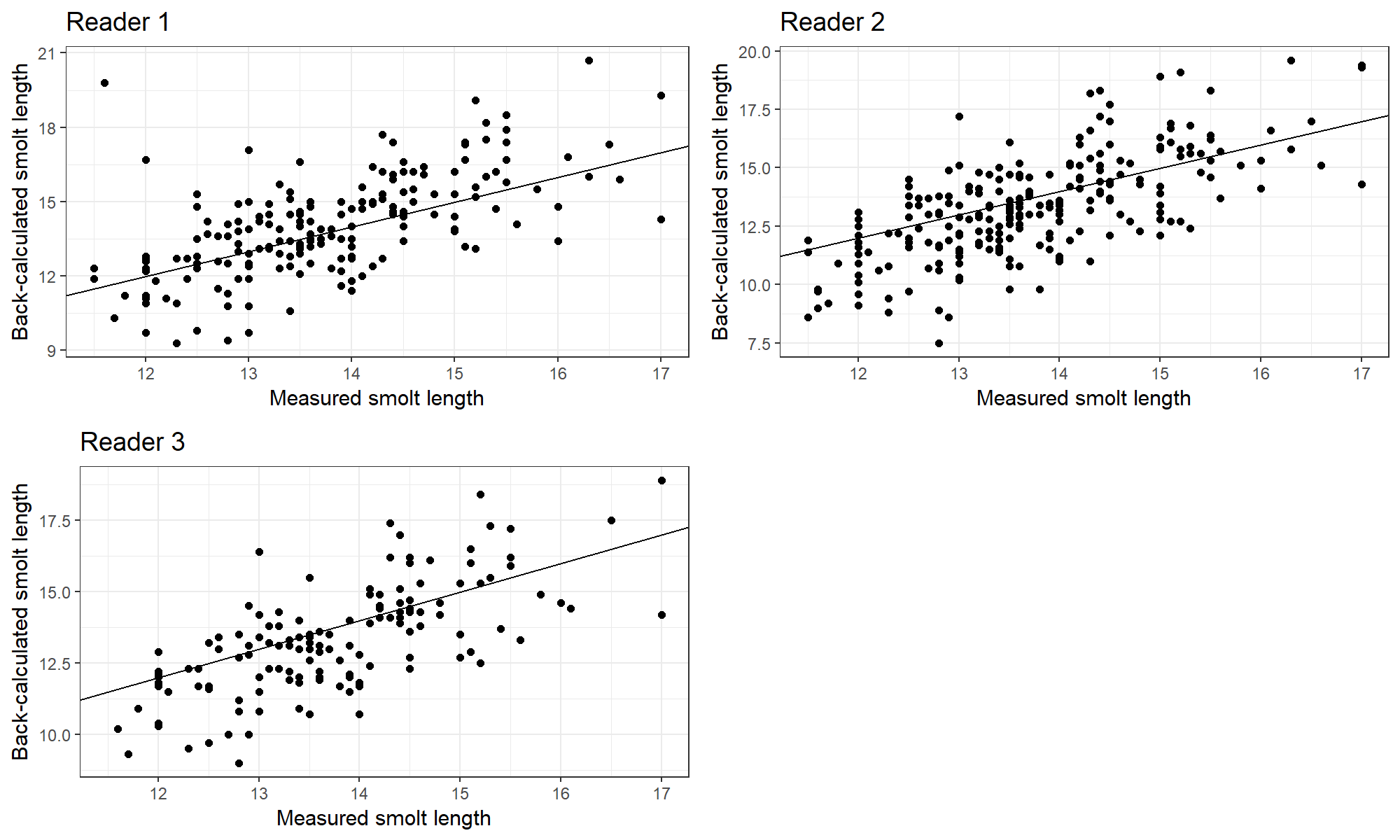

Supplement: S3 Fig — The relationship between measured smolt length (cm) and back-calculated smolt length (cm) by each of the three scale-readers. (TIFF) [file pone.0316075.s005.tiff]
