# Supplementary material for: Validating Atlantic salmon (Salmo Salar) scale reading by genetic parent assignment and PIT-tagging
Source: PLoS One. 2025 May 8;20(5):e0316075. doi: 10.1371/journal.pone.0316075 (PMC12061416; doi:10.1371/journal.pone.0316075)
Supplement: S1 Table — Minimum, maximum and average measured and back-calculated length growth. Measured growth is calculated as the difference in body-length between the length at return and smolt length, while back-calculated growth is the difference in back-calculated length at the end of a winter sone and smolt length. Year 1 represents the growth until the end of the first winter-zone, year 2 represents the plus growth gained during the spring before entering the river (for 1SW salmon) or until the end of the second winter zone (for MSW salmon), year 3 represents the plus growth gained during the spring before entering the river (for 2SW salmon) or to the end of third winter zone (for 3SW salmon). None of the 3SW salmon had plus growth the last spring at sea. (DOCX) [file pone.0316075.s002.docx]

Table S1. Minimum, maximum and average measured and back-calculated length growth. Measured growth is calculated as the difference in body-length between the length at return and smolt length, while back-calculated growth is the difference in back-calculated length at the end of a winter sone and smolt length. Year 1 represents the growth until the end of the first winter-zone, year 2 represents the plus growth gained during the spring before entering the river (for 1SW salmon) or until the end of the second winter zone (for MSW salmon), year 3 represents the plus growth gained during the spring before entering the river (for 2SW salmon) or to the end of third winter zone (for 3SW salmon). None of the 3SW salmon had plus growth the last spring at sea.

|  | Measured length growth (cm) | | | Average back-calculated growth (cm) | | |
| --- | --- | --- | --- | --- | --- | --- |
|  | Average | Min growth | Max | Year 1 | Year 2 | Year 3 |
| 1SW | 43.81 | 31.6 | 55.5 | 28.20 | 15.13 |  |
| 2SW | 64.42 | 42.0 | 81.0 | 28.55 | 27.80 | 7.01 |
| 3SW | 76.25 | 56.7 | 95.5 | 29.09 | 30.13 | 17.45 |
