# Supplementary material for: Validating Atlantic salmon (Salmo Salar) scale reading by genetic parent assignment and PIT-tagging
Source: PLoS One. 2025 May 8;20(5):e0316075. doi: 10.1371/journal.pone.0316075 (PMC12061416; doi:10.1371/journal.pone.0316075)
Supplement: S2 Table — The number of smolt lengths under- and overestimated by each of the three scale-readers, as well as χ2 and p-values estimated with the Evans Hoenig test. (DOCX) [file pone.0316075.s003.docx]

Table S2. The number of smolt lengths under- and overestimated by each of the three scale-readers, as well as χ^2^ and p-values estimated with the Evans Hoenig test.

|  | N under-est. | N over-est. | N-identical | χ^2^ | p-value |
| --- | --- | --- | --- | --- | --- |
| Reader 1 | 77 | 103 | 7 | 5.83 | 0.016 |
| Reader 2 | 145 | 93 | 2 | 10.417 | 0.001 |
| Reader 3 | 84 | 47 | 6 | 7.015 | 0.008 |
