# Supplementary material for: Validating Atlantic salmon (Salmo Salar) scale reading by genetic parent assignment and PIT-tagging
Source: PLoS One. 2025 May 8;20(5):e0316075. doi: 10.1371/journal.pone.0316075 (PMC12061416; doi:10.1371/journal.pone.0316075)
Supplement: Attachment — Submitted filename: Comments_20250115.docx [file pone.0316075.s006.docx]

**Manuscript: PONE-D-24-56358**

**Title: Validating Atlantic Salmon (*Salmo Salar*) Scale Reading by Genetic Parent Assignment and PIT-Tagging**

**General Comments:**

This paper provides an important dataset and addresses a critical question in fisheries science. However, to enhance its scientific rigor and broader applicability, the authors should improve the description of the experimental design, conduct a more in-depth and complete data analysis, and critically evaluate the implications of their findings. By addressing these issues, this manuscript has the potential to be a valuable contribution to the field.

**Specific Comments:**

1. The manuscript does not clearly articulate a hypothesis or research question. It is recommended that the authors explicitly state the hypothesis or objectives to provide a clearer research direction.
2. Provide detailed information on whether the sample size of 254 fish is sufficient to generalize the findings, either through statistical power analysis or reference to previous literature.
3. Include more detailed information on the distribution of fish characteristics, such as the precise sites of scale sampling, freshwater age, marine age, and size. A comprehensive description of these properties should be included in the Materials and Methods section.
4. Add images or diagrams to enhance reader understanding and provide clearer visualizations to highlight key findings and address potential biases.
5. Strengthen the manuscript by incorporating additional data, such as more detailed growth and environmental information for fish with known ages. Consider employing more robust validation methods, such as otolith analysis or independent genetic markers.
6. Provide details on controls or calibration methods used in the scale-reading process to minimize or control variability among readers.
7. Since the study relies heavily on experienced readers for scale analysis, include information on how their training was standardized. Discuss calibration procedures to ensure consistency and reliability among readers.
8. Elaborate on the steps taken to verify the accuracy of salmon back-calculated lengths using alternative methods or indicators.
9. Some claims made in the manuscript lack sufficient data support. For instance, the accuracy of Multi-Sea Winter (MSW) salmon is only briefly discussed. Expand on these findings with additional data or analysis.
10. Provide more detail about the statistical methods used. For example: Explain why generalized logistic regression was chosen and how it was validated. Justify the use of Pearson correlation analysis and discuss its limitations in this context.
11. The Discussion section mainly restates results without critically analyzing their implications. Include discussions on potential biases and limitations, such as reader variability or environmental factors affecting scale formation.
12. Situate the study within the broader context of fisheries science. Compare the findings with previous research on Atlantic salmon or other salmonids, highlighting similarities and differences.
